# Structurally Oriented Classification of *FOXA1* Alterations Identifies Prostate Cancers with Opposing Clinical Outcomes and Distinct Molecular and Immunologic Subtypes

**DOI:** 10.1158/1078-0432.CCR-24-3471

**Published:** 2025-01-02

**Authors:** Justin Hwang, Pornlada Likasitwatanakul, Sachin Kumar Deshmukh, Sharon Wu, Jason J. Kwon, Eamon Toye, David Moline, Mark G. Evans, Andrew Elliott, Rachel Passow, Christine Luo, Emily John, Nishant Gandhi, Rana R. McKay, Elisabeth I. Heath, Chadi Nabhan, Natalie Reizine, Jacob J. Orme, Josep M. Domingo Domenech, Oliver Sartor, Sylvan C. Baca, Scott M. Dehm, Emmanuel S. Antonarakis

**Affiliations:** 1 Masonic Cancer Center, University of Minnesota-Twin Cities, Minneapolis, Minnesota.; 2 Department of Medicine, University of Minnesota-Twin Cities, Minneapolis, Minnesota.; 3 Dana Farber Cancer Institute, Boston, Massachusetts.; 4 Department of Medicine, Siriraj Hospital, Mahidol University, Bangkok, Thailand.; 5 Department of Medical Affairs, CarisLifeSciences, Irving, Texas.; 6 Broad Institute of MIT and Harvard, Cambridge, Massachusetts.; 7 Perelman School of Medicine, University of Pennsylvania, Philadelphia, Pennsylvania.; 8 University of California San Diego, San Diego, California.; 9 Karmanos Cancer Institute, Wayne State University, Detroit, Michigan.; 10 University of Illinois at Chicago, Chicago, Illinois.; 11 Mayo Clinic, Rochester, Minnesota.

## Abstract

**Purpose:**

Around 10% to 15% of prostate cancers harbor recurrent aberrations in the Forkhead Box A1 gene, *FOXA1*, whereby the alteration type and the effect on the forkhead (FKH) domain affect protein function. We developed a *FOXA1* classification system to inform clinical management.

**Experimental Design:**

A total of 5,014 prostate cancer samples were examined using whole-exome and -transcriptome sequencing from the Caris Life Sciences database. We denoted class 1 *FOXA1* alterations as missense and in-frame insertions/deletions with subclasses oriented with respect to the FKH domain. These were in the first part of the FKH domain [class 1A: amino acids (AA) 168–246], within the Wing2 region of FKH (class 1B: AA 247–269), or outside FKH (class 1C: AA 1–167, 270+). Two hotspot missense mutations at R219 were denoted class 2. Class 3 included predicted truncating mutations with subclasses partitioned based on the FKH domain (class 3A: AA 1–269 and class 3B: AA 270+). Class 4 represented *FOXA1* amplifications. Real-world overall survival and therapy outcomes were determined from insurance claims.

**Results:**

*FOXA1* alterations did not influence survival when considered in aggregate but had distinct prognostic effects when stratified by class. In primary prostate samples, class 1A alterations were associated with overall improved survival (HR, 0.57; *P* = 0.03); a similar trend was seen in metastatic biopsies with class 1B (HR, 0.84; *P* = 0.09). Conversely, in primary specimens, class 1C exhibited worse survival upon second-generation androgen receptor signaling inhibitor treatment (HR, 1.93; *P* < 0.001). Class 2 mutations (R219C/S) were enriched in neuroendocrine prostate cancers and were associated with overall poor survival (HR, 2.05; *P* < 0.001) and worse outcomes to first-line androgen-deprivation therapies (HR, 2.5; *P* < 0.001). Class 3A alterations indicated improved survival (HR, 0.70; *P* = 0.01), whereas class 3B alterations portended poor outcomes (HR, 1.50; *P* < 0.001). Amplifications (class 4) indicated poor outcomes in metastatic samples (HR, 1.48; *P* = 0.02). Molecularly, different *FOXA1* alteration classes harbored distinct mutational and immunologic features as well as unique transcriptional programs. Finally, relative to European Americans, African Americans had increased class 1C alterations, whereas Asian/Pacific Islander patients had increased class 1B alterations.

**Conclusions:**

*FOXA1* alterations should not be interpreted in aggregate, as different classes are associated with divergent molecular features and clinical outcomes. Our revised classification schema facilitates clinical decision-making for patients with prostate cancer and uncovers important racial differences.

**Translational Relevance**

We propose a clinically relevant revised *FOXA1* classification scheme for patients with prostate cancer that includes four main classes and seven subclasses based on all *FOXA1* alterations observed in 191,586 solid tumor specimens from the Caris Life Sciences Precision Oncology Alliance database. In the 5,014 prostate tumors, these classes are associated with different survival outcomes (some improving survival and some worsening survival) and are disproportionally represented across races. These alteration classes capture tumors that are characterized by distinct somatic drivers, signaling pathways, and tumor-immune microenvironments. Given that many clinical-grade genomic tests now report *FOXA1* aberrations, our work urges the evaluation of *FOXA1* alteration classes individually (rather than in aggregate) when managing patients with advanced prostate cancer.

## Introduction

Prostate cancer is the most prevalent noncutaneous cancer in men and results in more than 250,000 deaths worldwide (1). The androgen receptor (AR) is a protumorigenic transcription factor, and aberrant AR signaling drives metastasis and resistance to androgen-deprivation therapy (ADT), a mainstay for prostate cancer treatment (2). Currently, deciphering ADT response and improving targeted therapeutics remain critical avenues to improve outcomes for patients with prostate cancer.

In prostate tumor cells, the AR binds essential cofactors including FOXA1 (3), which functions as a pioneering and transcription factor (4, 5). In late-stage disease, FOXA1 can “reprogram” AR activity (6, 7), thereby promoting tumor progression, lineage plasticity, and therapy resistance (6–9). High *FOXA1* expression is thought to be associated with poor survival, increased AR expression, higher serum PSA, and higher Gleason grades (10, 11). FOXA1 may also play a role in neuroendocrine prostate cancer (NEPC). At a mutation prevalence of 10% to 15%, *FOXA1* is one of the most frequently altered genes in prostate cancer (8, 12, 13). The majority of *FOXA1* alterations are missense mutations or in-frame insertions/deletions (indel) that affect the forkhead (FKH) protein domain [amino acids (AA) 168–269; refs. 8, 9]. The majority of FKH domain alterations are observed in the Wing2 region (AA 248–269; refs. 8, 9, 14), which regulates DNA binding and transcription (15, 16). However, current clinical-grade genomic tests do not always report *FOXA1* alterations with an eye toward guiding treatment decisions. Therefore, a reliable framework to detect and characterize *FOXA1* alterations may improve outcomes for patients with advanced prostate cancer (17).

In this study, using prior classifications of *FOXA1* alterations as our guide (8, 9), we devised a revised classification scheme to stratify patients with prostate cancer based on different types of *FOXA1* alterations using a cohort of 5,014 prostate tumors subjected to whole-exome and -transcriptome next-generation sequencing (NGS). This revised nomenclature yielded four main classes and seven subclasses of *FOXA1* alterations, as well as a wild-type (WT) group. We then tested a hypothesis comparing the *FOXA1* WT group with each altered group to determine whether specific classes of *FOXA1* alterations have divergent molecular, immunologic, and clinical characteristics.

## Materials and Methods

### Clinical information and survival analysis

We queried the deidentified real-world evidence outcomes dataset from the Caris Life Sciences Precision Oncology Alliance (POA) registry. Real-world evidence overall survival (OS) was defined as the time from the date of treatment initiation to either death or the last contact extracted from insurance claim data, as previously validated (18). Patient death was assumed for any patient without a claim for more than 100 days, which holds true for more than 95% of patients with a recorded death in the National Death Index. HR was calculated using the Cox proportional hazards model, and *P* values were calculated using the log-rank test. Treatment information was derived from insurance claim data that were submitted to the Caris Life Sciences database. Treatments may occur at any point along the cancer progression, and OS was calculated from the time of treatment initiation to either death or the time of the last patient contact. To account for castration status, we considered all systemic therapies started within 90 days of initial first-line ADT to represent the hormone-sensitive (noncastrate) setting, whereas systemic therapies started >90 days after first-line ADT were denoted as castration-resistant treatments, as previously described (19). This study was conducted in accordance with the guidelines of the Declaration of Helsinki, Belmont report, and U.S. Common Rule. In keeping with 45 CFR 46.101(b)32, this study was performed using retrospective, deidentified clinical data. Therefore, this study is considered exempt from institutional review board approval, and no patient consent was necessary from the subjects. No animal studies were conducted in our research.

### Pathologic classification

Specimens submitted to Caris Life Sciences were reviewed by an in-house genitourinary pathologist. Prostate cancer subtypes were defined by the World Health Organization classification of tumors, and morphologic analysis for adenocarcinomas versus small-cell/neuroendocrine carcinomas was conducted according to consensus criteria (20).

### Sample purification

In order to increase the tumor density of samples undergoing DNA/RNA extraction for NGS, microdissection was performed on all tumor samples, and the area of slides with the highest concentration of cancer cells was separated from the area of normal cells. Specifically, hematoxylin and eosin–stained slides were reviewed under a light microscope by a pathologist, and the tumor area was marked. Slides designated for microdissection were then stained with a nuclear fast red stain, and the tumor area of the stained slides was then manually dissected accordingly for DNA/RNA extraction. A minimum of 10% tumor content in the area for microdissection was required to enable the enrichment and extraction of tumor-specific DNA/RNA.

### NGS platform

NGS was performed on genomic DNA isolated from formalin-fixed, paraffin-embedded tumor samples using the NextSeq or NovaSeq 6000 platform (Illumina, Inc.) in a Clinical Laboratory Improvement Amendments/College of American Pathologists/International Organization for Standardization certified clinical laboratory (Caris Life Sciences). For NextSeq-sequenced tumors, a custom-designed SureSelect XT assay was used to enrich 592 whole-gene targets (Agilent Technologies). For whole-exome sequencing, a hybrid pulldown of baits designed to enrich for 720 clinically relevant genes at 1,500 × high coverage and read-depth was used, along with another panel designed to enrich for an additional >20,000 genes at a lower depth. A 500-Mb SNP backbone panel (Agilent Technologies) was added to determine copy-number alterations. The internal control step includes the normalization of the sequencing depth of each exon divided by the average sequencing depth within the sample and comparing the copy number of genes with the precalibrated mean of normalized values in the training data. Furthermore, the mean values are recalibrated every 60 days with up to 10,000 samples across all cancer types. Gene amplification is then defined as ≥6 copies.

### Interpretation of genetic variants

Genetic variants were interpreted by board-certified molecular geneticists and classified as pathogenic, likely pathogenic, variants of unknown significance, likely benign, or benign according to the American College of Medical Genetic and Genomics standards (RRID: SCR_005769). When assessing the mutation frequencies of individual genes, pathogenic and likely pathogenic alterations were counted as mutations, whereas benign, likely benign, and variants of unknown significance were excluded. High tumor mutational burden (TMB) was defined as tumors with ≥10 mutations/megabase of DNA.

### Whole-transcriptome sequencing

The Qiagen RNA FFPE tissue extraction kit was used for extraction, and RNA quality and quantity were determined using the Agilent TapeStation system (RRID: SCR_018435). Biotinylated RNA baits were hybridized and then amplified through a post-capture PCR reaction. Raw data were demultiplexed using the Illumina Dragen Bio-IT accelerator, trimmed, counted, removed of PCR duplicates, and aligned to the human reference genome hg19 by STAR aligner (RRID: SCR_004463). For transcription counting, transcripts per million molecules were generated using the Salmon expression pipeline.

### Missense tolerance ratio analyses

Missense tolerance ratio (MTR) scores were obtained through the analysis of exomes and genomes (21). Binomial exact tests assessed variations in observed missense mutation rates at each AA residue, with results adjusted for FDR using the Benjamini–Hochberg method as previously described (22).

### Structure visualization and *in silico* ddG analyses

The crystal structure of FOXA1 (PDB: 7VOX) was visualized using PyMOL (RRID: SCR_000305; refs. 23, 24). *In silico* saturation mutagenesis on *FOXA1* was performed using FoldX (RRID: SCR_008522), and MutateX was used for automation on the structural file (25).

### Structure prediction of FOXA1 using AlphaFold

The FOXA1 protein sequence (UniProt ID: P55317-1, RRID: SCR_002380; refs. 26) was used as the WT reference. Two additional FOXA1 sequences were created by manually altering position R219 into R219C and R219S variants. Structural predictions for each sequence were done using AlphaFold2 (2.3.1-multimer; RRID: SCR_023662; ref. 27). The model with the highest average predicted local distance difference test score for each sequence was used for visualization using PyMOL (2.5.0; RRID: SCR_000305). The forehead domain (AA 168–269) for each sequence was isolated and then aligned using the “super” command to superimpose them and calculate the root mean square deviation. A ray-traced image (ray_trace_mode, 1; ray_trace_color, black; ray_opaque_background, off; and ray_shadows, 0) centered on residue 219 was created to compare the WT R219 residue with the mutated R219C and R219S residues alongside their superimposed positions.

### Gene expression analysis

IFNγ scores (28) and T-cell inflammation scores (29) were calculated using priors signatures. The AR and NEPC signatures were calculated from Beltran and colleagues (30). The Gene Set Enrichment Analysis (GSEA; RRID: SCR_003199; ref. 31) of the hallmarks of cancer collection was used to evaluate pathway enrichment between FOXA1 mutation classes and WT tumors.

### Immune cell deconvolution

Immune cell tumor infiltration was inferred using quanTIseq (RRID: SCR_022993; ref. 32) based on the deconvolution of bulk RNA sequencing data as previously described.

### Genomic associations

The frequency of associated somatic genetic mutations in each *FOXA1* subclass was calculated with pairwise statistics of either the Fisher exact test or *χ*^2^ test. *q* value < 0.05 is used to determine the significance level.

### Data availability

The principal investigator (E.S. Antonarakis) has full access to all the data in this study and takes responsibility for the integrity of the data and the accuracy of the data analysis. The data are not publicly available due to data size and patient privacy considerations but are available upon reasonable request through a Caris letter of intent (www.carislifesciences.com/letter-of-intent).

## Results

### Prostate cancer carries the highest *FOXA1* alteration frequency of all cancers

**Figure 1.**
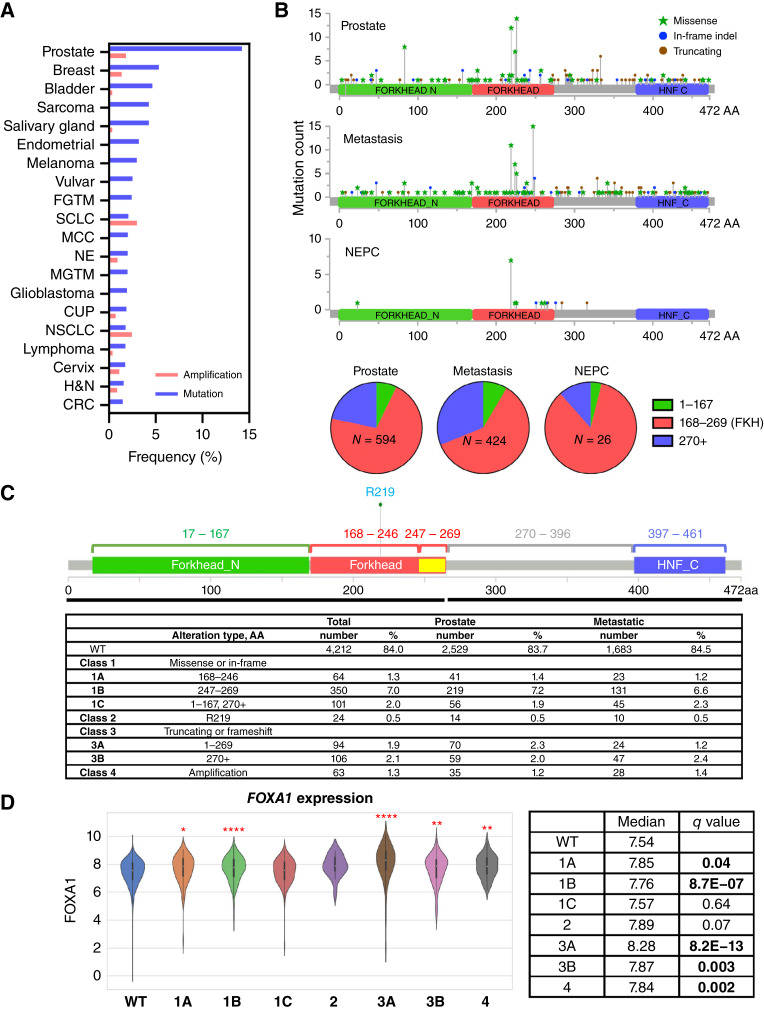
*FOXA1* alterations in prostate cancer. **A,** Bar graphs showing percentages of amplifications and mutations found in the top 20 cancers with *FOXA1* alterations from the Caris Life Sciences POA database. CUP, carcinoma of unknown primary; CRC, colorectal cancer; FGTM, female genital tract malignancy; H&N, head and neck; MCC, Merkel cell carcinoma; MGTM, male genital tract malignancy; NE, neuroendocrine; NSCLC, non–small cell lung cancer; SCLC, small cell lung cancer. **B,** Lollipop plots showing different types of alterations along the *FOXA1* gene body in primary prostate, metastatic, and histologically defined NEPC samples. The mutations are summarized by AA residues in the pie chart. HNF_C, hepatocyte nuclear factor C terminus domain. **C, ***FOXA1* alteration classification used in this study and the table showing numbers and percentages of each classification in our cohort. **D,** Violin plots showing *FOXA1* mRNA expression based on alteration class. The median is reflected by horizontal white lines. *q* values are established based on comparisons with WT. *, *q* value < 0.05; **, *q* value < 0.01; ***, *q* value < 0.001; ****, *q* value < 0.0001.

Using the Caris Life Sciences POA database, we examined the alterations of *FOXA1* in 20 cancer types encompassing 191,586 solid tumors (Supplementary Table S1). Prostate cancer exhibited the greatest proportion of *FOXA1* alterations, with 14.2% of tumors harboring mutations and 1.8% showing amplifications (Fig. 1A). After prostate cancer, 6.7%, 5.0%, and 4.6% of breast, bladder, and salivary cancers harbored *FOXA1* alterations. This finding suggested that *FOXA1* mutations may be enriched in hormonally driven cancers.

### *FOXA1* alterations in prostate cancer exhibit recurrent mutational patterns

Through the interrogation of 5,014 prostate cancer samples (Table 1), we found that *FOXA1* alterations could be subdivided into three broad mutational mechanisms: missense mutations or in-frame indels, predicted truncating events, or gene amplifications. We mapped *FOXA1* alterations found in primary prostatic tumors, metastatic sites, or histologic NEPC based on the protein domains (Fig. 1B). *FOXA1* alterations predominantly clustered in the FKH domain (Supplementary Fig. S1). More missense alterations were found before or within the FKH domain, whereas alterations after the FKH domain were predominantly truncating events. Within the FKH domain, the majority of *FOXA1* alterations were within the Wing2 region, confirming several hotspots previously described (8, 9). Notably, missense mutations involving residue R219 were strikingly enriched in NEPC samples relative to adenocarcinomas. Furthermore, when we examined tumor samples across all 20 cancer types, we noted that these R219 mutations were pathognomonic for prostate cancer.

**Table 1.** Patient demographics.

Patient characteristic	Total	*FOXA1* altered	*FOXA1* WT
Number of cases	5,014	802 (16%)	4,212 (84%)
Age, years [median (range)]	68 (35–90)	70 (41–90)	68 (35–90)
Race, *N* (%)			
AP	126 (3.01)	32 (4.77)	94 (2.68)
Black/AF	722 (17.3)	130 (19.4)	592 (16.9)
White/EA	3,126 (74.7)	479 (71.4)	2,647 (75.4)
Other	209 (5)	30 (4.47)	179 (5.1)
Ethnicity, *N* (%)			
Hispanic or Latino	420 (10.5)	60 (9.66)	360 (10.6)
Not Hispanic or Latino	3,587 (89.5)	561 (90.3)	3,026 (89.4)
Site of biopsy, *N* (%)			
Prostate gland	3,023 (60.3)	494 (61.6)	2,529 (60)
Metastatic site	1,991 (39.7)	308 (38.4)	1,683 (40)
Lymph node (% of metastases)	622 (31.2)	95 (30.8)	527 (31.3)
Bone (% of metastases)	355 (17.8)	58 (18.8)	297 (17.6)
Liver (% of metastases)	291 (14.6)	45 (14.6)	246 (14.6)
Other (% of metastases)	723 (36.3)	110 (35.7)	613 (36.4)
Histology, *N* (%)			
Adenocarcinoma	4,901 (97.7)	778 (97)	4,123 (97.9)
NEPC/small-cell cancer	113 (2.25)	24 (3)	89 (2.11)
Type of sequencing			
WES only	1,156	178	978
WES and WTS	3,858	624	3,234
Immune markers, *N* (%)			
MSI-high	189 (3.79)	52 (6.51)	137 (3.28)
TMB-high	189 (3.92)	51 (6.72)	138 (3.4)
Treatments	4,803	758	4,045
First-generation ADT	3,398	533	2,865
Second-generation ARSI	2,382	371	2,011
Taxane chemotherapy	1,590	250	1,340

Abbreviations: WES, whole-exome sequencing; WTR, whole-transcriptome sequencing.

### Structurally informed revised classification of *FOXA1* alterations

We next devised a classification system to partition *FOXA1* mutations into four classes and seven subclasses (Fig. 1C). Class 1 alterations included missense or in-frame indel mutations. Mutations that occurred inside the FKH domain (AA 168–269) were further classified into those that occurred in the first portion of the FKH domain (AA 168–246) and those in the Wing2 region (AA 247–269) as classes 1A and 1B, respectively. Missense/in-frame mutations outside the FKH domain were assigned as class 1C. Noting the R219S/C mutation enrichment in NEPC, we separately assigned these as class 2. Class 3 events were the predicted truncating mutations, with 3A denoting truncations at AA 1 to 269 (in or before the FKH domain), whereas 3B included truncations at or after AA 270 (after the FKH domain). Finally, *FOXA1* amplifications were denoted as class 4. Unexpectedly, we observed no enrichment of any *FOXA1* alteration classes in metastatic samples relative to primary samples (Fig. 1C). However, when examining *FOXA1* class 1 to 3 alterations based on the variant allele frequency, we noted that the median variant allele frequency of each class was greater in metastatic tumors (Supplementary Fig. S2A and S2B). To determine the effect of each alteration class on *FOXA1* gene expression, we characterized the median transcript levels in each class. Interestingly, class 1A, 1B, 3A, 3B, and 4 alterations were each associated with significant overexpression of *FOXA1* transcripts (Fig. 1D), suggesting that these mutations are activating rather than inactivating. Conversely, we found that different *FOXA1* classes were associated with only modest changes in *AR* expression or when we interrogated AR or NEPC signatures by class type (Supplementary Fig. S3A–S3C).

**Figure 2.**
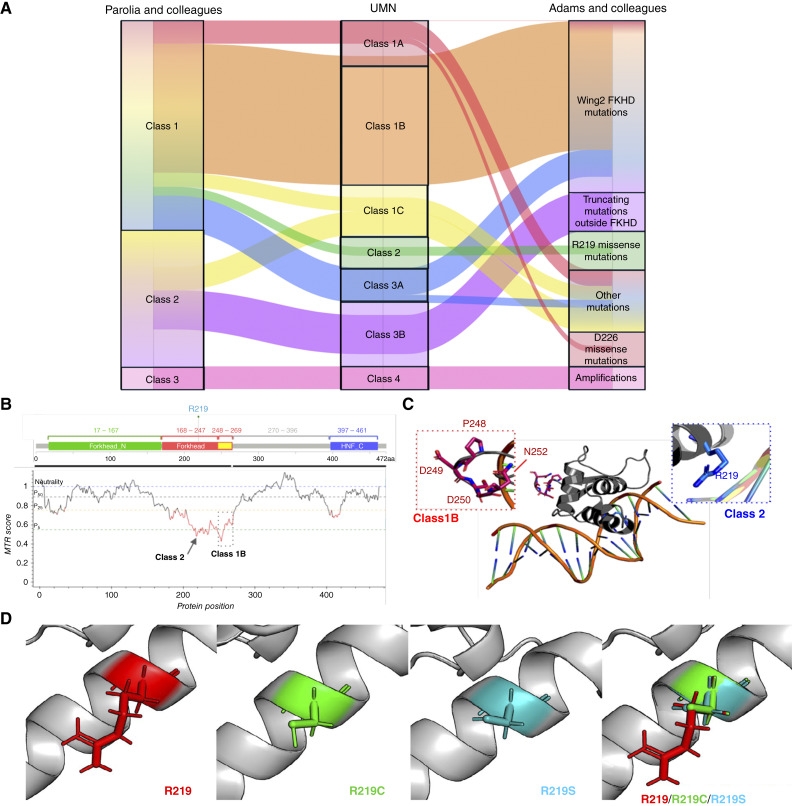
*FOXA1* alterations and implications on protein structure. **A,** Distinctions between our classification scheme and two seminal studies are shown through an alluvial plot. **B,** MTR of each AA position of FOXA1. Horizontal lines show gene-specific MTR percentile: 5th (green), 25th (yellow), 50th (black), and neutrality (blue, MTR = 1.0). HNF_C, hepatocyte nuclear factor C terminus domain. **C,** Structural overview of class 1B and class 1C alterations on FOXA1 structure with AA of each class represented as sticks. **D,** The FKH domain was modeled using AlphaFold2, and the consequences of point mutations in R219C/S are visually depicted. UMN, University of Minnesota.

We next compared our University of Minnesota classification scheme to the two seminal studies by Adams and colleagues (8) and Parolia and colleagues (Fig. 2A; ref. 9), who previously classified *FOXA1* by integrating distinct cohorts. The University of Minnesota classification displayed many overlapping features with the two prior *FOXA1* classifications while also having some distinct components.

### Class 1B and 2 residues are essential to WT FOXA1 function

We next sought to understand the impact of class 1B and 2 mutations on FOXA1 protein function by studying regional intolerance to particular AA variations. MTR scoring estimates the impact of genetic changes on protein functionality (i.e., whether a protein can “tolerate” AA changes without compromising its structure/function). We leveraged MTR scoring to evaluate the relative essentiality of each AA residue in FOXA1 and found that class 1B (AA 247–269) and class 2 (R219) residues had notably lower MTR scores, indicating high functional importance (Fig. 2B). Examining by class, class 1B and 2 mutations had lower MTR scores compared with class 1C or 1A alterations (Supplementary Fig. S4A). We also performed *in silico* mutagenesis studies based on the known 3D structure of the FKH domain (24). The average free-energy change (ΔΔG) in intrinsic stability for class 1B and 2 mutations was significantly higher than that in WT FKH by 1.20 and 1.39 kcal/mol, respectively, compared with class 1A mutations at 0.15 kcal/mol (Supplementary Fig. S4B). Based on the previously resolved structure of the FOXA1 FKH domain (21, 22), class 1B residues fell within the flexible loop, whereas the class 2 residue (R219) engaged the DNA-binding helix (Fig. 2C). In prostate cancer, class 1B and 2 alterations likely regulate different but essential processes for tumor homeostasis, which may explain why they are enriched despite being energetically unfavorable for the cancer cell. Finally, we used AlphaFold2 (27) to model the potential consequences on the α-helix domain of FKH (Fig. 2D), which indicated that R219C/S alterations did not alter the overall α-helix structure, but the cysteine and serine side chains were visually distinct from the side chains based on WT arginine in this modeling approach. Taken together, these data suggest that class 1B and 2 *FOXA1* alterations are structurally and functionally distinct from all other alteration classes and from each other.

### *FOXA1* alteration classes are associated with opposing clinical outcomes

**Figure 3.**
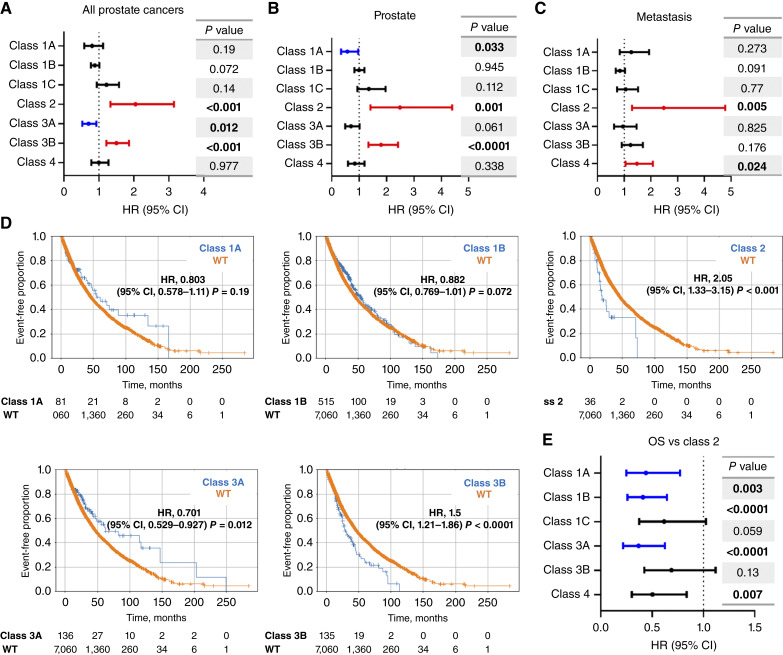
OS based on *FOXA1* alteration class. OS is depicted based on forest plots as organized by each *FOXA1* alteration class for (**A**) all prostate tumors, (**B**) prostate samples, and (**C**) metastatic samples. **D,** Kaplan–Meier curves for different alterations classes compared with WT. **E,** Outcomes for class 2 are compared with all other alteration classes.

To estimate the effects on survival, we first examined the aggregate of all *FOXA1* alterations and compared this with WT samples. When doing so, using either all tumors or those acquired from the prostate or metastasis (Supplementary Fig. S5), *FOXA1*-altered samples overall did not exhibit significant survival differences as compared with WT. However, upon applying our classification scheme, we noted many differences in OS that informed either better or worse OS (Fig. 3A–D; Supplementary Table S2). Relative to WT, class 2 was consistently associated with poor OS in all tumors [HR, 2.05; 95% confidence interval (CI), 1.33–3.15; *P* < 0.001] and when considering primary or metastatic tumors separately (HR, 2.49 and 2.48; 95% CI, 1.41–4.40 and 1.29–4.78; *P* = 0.001 and 0.005, respectively). Classes 3A and 3B, although both classified by truncating/frameshift mutations, informed of opposing outcomes in all tumors and in primary tissue biopsies. In all tumors, class 3A indicated improved OS (HR, 0.70; 95% CI, 0.53–0.93; *P* = 0.012), whereas class 3B was associated with worse OS (HR, 1.50; 95% CI, 1.21–1.86; *P* < 0.0001). In primary tumor biopsies, we again observed this opposing trend for classes 3A (HR, 0.70; 95% CI, 0.49–1.02; *P* = 0.061) and 3B (HR, 1.80; 95% CI, 1.34–2.42; *P* < 0.0001). Class 1A was associated with improved OS in prostate biopsies (HR, 0.57; 95% CI, 0.335–0.961; *P* = 0.033), whereas class 4 indicated poor outcomes in metastatic samples (HR, 1.48; 95% CI, 1.05–2.07; *P* = 0.02). Furthermore, when directly comparing class 2 alterations with other *FOXA1* classes, all the other classes exhibited better OS with classes 1A, 1B, and 3A being statistically significant (Fig. 3E).

We also conducted further pathologic review of the 24 samples with class 2 mutations (Supplementary Tables S3 and S4) and determined that 16 were adenocarcinomas morphologically (66.6%), whereas eight (33.3%) were nonadenocarcinomas (five small-cell prostate cancers and three NEPCs; ref. 20); 14 of the 16 *FOXA1* class 2–mutant adenocarcinomas were from metastatic sites. This indicated that although R219 missense mutations may be enriched in NEPC, they can also be concealed within pure acinar adenocarcinoma cases. This finding increases the clinical importance of this observation because it uncovers a negative prognostic feature in prostatic adenocarcinomas that have no histologic evidence of neuroendocrine/small-cell morphology.

**Figure 4.**
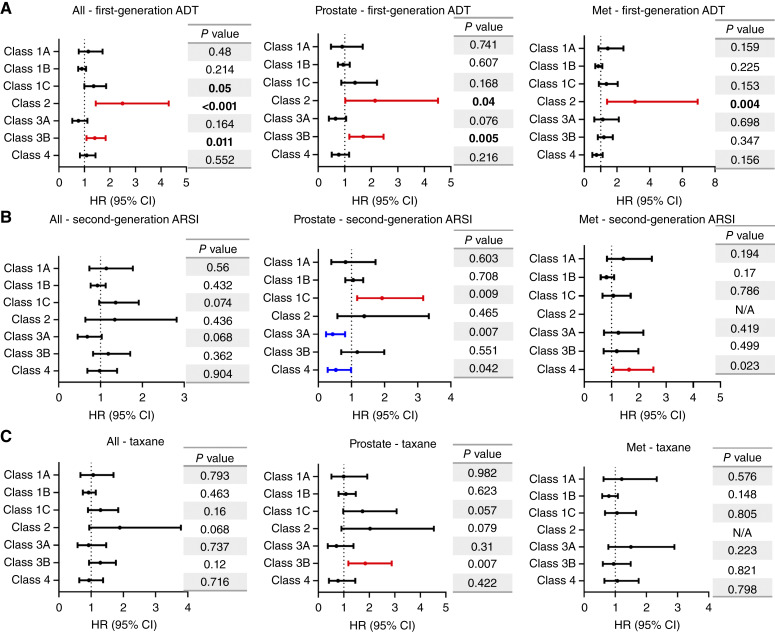
Treatment-associated outcomes by *FOXA1* alteration class. The survival analysis is conducted on patients who received (**A**) first-line ADT, including goserelin, leuprolide, triptorelin, degarelix, and relugolix. **B,** Second-generation ARSI, including apalutamide, abiraterone, darolutamide, and enzalutamide. **C,** Taxane chemotherapy, including docetaxel or cabazitaxel. N/A, not available.

We next considered how each class of *FOXA1* alteration associated with outcomes based on the first instance of prostate cancer systemic treatment by first-line ADT, second-generation AR signaling inhibitors (ARSI), and taxane chemotherapies (Fig. 4A–C; Supplementary Table S5). In this study, the classes of *FOXA1* alterations exhibited many prognostic differences. Class 1C alterations exhibited worse outcomes in prostate biopsies treated with second-generation ARSI (HR, 1.93; 95% CI, 1.17–3.17; *P* = 0.009). Class 2 exhibited worse outcomes to first-generation ADT in both prostate and metastatic samples (HR, 2.15, 3.11; 95% CI, 1.02–4.52, 1.39–6.94; *P* = 0.04, 0.004, respectively) and exhibited trends toward worse outcomes in prostate cancers treated with taxanes (HR, 2.03; 95% CI, 0.905–4.53; *P* = 0.079). In primary prostate tumors, class 3A exhibited trends toward improved response to first-generation ADT and significant differences to second-generation ARSI (HR, 0.650, 0.433; 95% CI, 0.402–1.051, 0.231–0.809; *P* = 0.076, 0.007, respectively). Class 3B again was distinct from 3A and indicated worse outcomes in primary prostate tumors treated with first-generation ADT and taxanes (HR, 1.70, 1.84; 95% CI, 1.18–2.88, 0.99–2.42; *P* = 0.005, *P* = 0.007). Interestingly, class 4 alterations exhibited opposing outcomes in primary and metastatic samples treated with second-generation ARSI (HR, 0.529, 1.64; 95% CI, 0.283–0.987, 1.07–2.54; *P* = 0.042, 0.023, respectively).

Among the TMB-high and microsatellite instability (MSI)-high patients, we examined patient subsets that also harbored concurrent *FOXA1* alterations (Supplementary Table S6). Based on the limited numbers, TMB-high or MSI-high tumors with *FOXA1* coalterations that were treated with on-label pembrolizumab had improved outcomes relative to those with TMB-high or MSI-high status alone (HR, 0.399, 0.338; 95% CI, 0.169–0.941, 0.119–0.965; *P* = 0.03, 0.034; Supplementary Fig. S6). In this study, we also confirmed that the TMB-high and MSI-high tumors had increased alteration frequencies of mismatch repair genes including *MLH1*, *MSH2/6*, and *PMS2* as compared with TMB-low and microsatellite-stable tumors (Supplementary Table S7), as expected.

### Divergent molecular features among classes of *FOXA1* alterations

**Figure 5.**
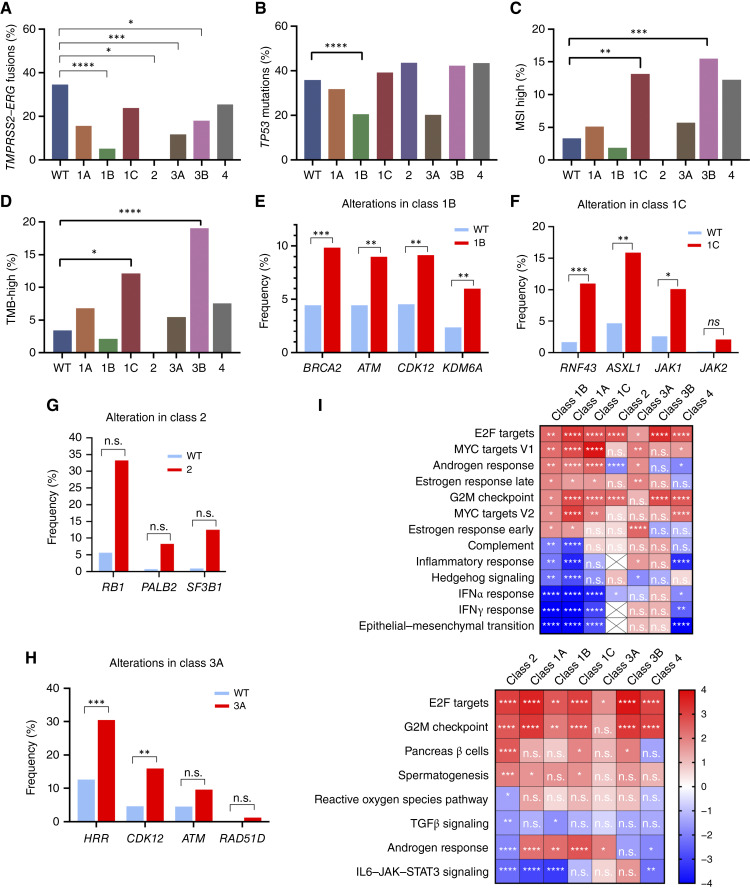
Molecular associations by *FOXA1* alteration class. Percentages of (**A**) *TMPRSS2–ERG* fusions, (**B**) *TP53* mutations, (**C**) MSI-high status, and (**D**) TMB-high status based on *FOXA1* alteration classes. All *q* values are relative to WT cases. Percentages of select genetic alterations comparing WT and (**E**) class 1B, (**F**) class 1C, (**G**) class 2, (**H**) and class 3A. *, *q* value < 0.05; **, *q* value < 0.01; ***, *q* value < 0.001; ****, *q* value < 0.0001. **I,** Heatmaps are organized by hallmark pathways that are significantly enriched (FDR <0.05) in class 1B and 2 in which normalized enrichment scores are depicted. *, FDR < 0.05; **, FDR < 0.01; ***, FDR < 0.001; ****, FDR < 0.0001. HRR, homologous recombinant repair genes; ns, nonsignificant.

We next examined the whole exomes and transcriptomes to identify class-specific molecular differences (Fig. 5; Supplementary Figs. S6–S9; Supplementary Tables S8 and S9). Classes 1B, 2, 3A, and 3B each exhibited significantly fewer *TMPRSS2*–*ERG* fusions, with 1B and 2 tumors essentially devoid of *TMPRSS2*–*ERG* fusions compared with a 34.5% prevalence in WT cases (Fig. 5A). Class 1B was depleted in pathogenic *TP53* mutations (20.4% vs. 35.8%; Fig. 5B). Only a small proportion of prostate cancers are typically MSI- or TMB-high, two markers that may inform the use of immune checkpoint therapies (33, 34). Relative to WT tumors (at 3.3% and 3.4%, respectively), class 1C (13.1% and 12.1%) and class 3B (15.5% and 19.0%) alterations demonstrated four- to fivefold greater rates of MSI- and TMB-high tumors (Fig. 5C and D), possibly suggesting that these alterations may be epiphenomena of the hypermutation process. Furthermore, the different classes of *FOXA1* alterations had different degrees of associations with other somatic gene mutations in the homologous recombinant repair and other pathways (Fig. 5E–H). These results exhibited similar trends even when partitioned into prostate and metastatic samples (Supplementary Figs. S7A–S7H and S8A–S8H).

We subsequently analyzed the effects of each class of *FOXA1* alteration on tumor transcriptomes. All classes except class 1C led to significant changes in gene expression profiles (Supplementary Fig. S9; Supplementary Table S9). Notably, although class 1A, 1B, 1C, and 2 alterations were all missense mutations or indels, these classes exhibited notable differences in their respective transcriptomes. Furthermore, although class 3A and 3B alterations were defined by protein-truncating lesions, they had different effects on their transcriptomes. Altogether, our genomic and transcriptomic data indicate that the different classes of *FOXA1* alterations exhibited divergent molecular profiles.

Finally, based on GSEA analysis (31), the classes of *FOXA1* alterations were differentially enriched in many hallmark RNA expression pathways (Fig. 5I). Notably, the androgen response pathway was elevated in classes 1A, 1B, 1C, and 3A and expectedly decreased in class 2, which included NEPC samples. All classes except class 3A exhibited enrichment in cell cycle–related mechanisms (G2M and E2F). Of note, classes 1A and 1B, both consisting of missense mutations in FKH, were depleted of epithelial-to-mesenchymal transition, as well as inflammatory response and IFNα/γ pathways. This suggested that FKH domain mutations in *FOXA1* do not exhibit lineage plastic profiles that may affect the tumor microenvironment (TME).

### Class 1B tumors possess distinct TME

We also explored how *FOXA1* alteration classes influenced mRNA-based immunologic signatures as well as inferred immune cell densities. Class 1B and 4 tumors exhibited reduced IFN-γ signaling, indicating that they have overall lower immune activation (Supplementary Fig. S10A). Furthermore, based on the T-cell inflammation score by sample, class 1A and 1B tumors had reduced T-cell–associated inflammation (Supplementary Fig. S10B). We next used quanTIseq to deconvolute immune cell fractions associated with all tumors (32). Class 1B mutations were associated with a unique TME, exhibiting decreases in regulatory T cells (Supplementary Fig. S10C), CD8 T cells (Supplementary Fig. S10D), and dendritic cells (Supplementary Fig. S10E) but increases in neutrophils (Supplementary Fig. S10F) and B cells (Supplementary Fig. S10G). Taken together, these findings indicate that class 1B *FOXA1* alterations are associated with an immunosuppressed milieu relative to *FOXA1* WT patients (Supplementary Table S10).

### Race-based differences in *FOXA1* alterations

**Figure 6.**
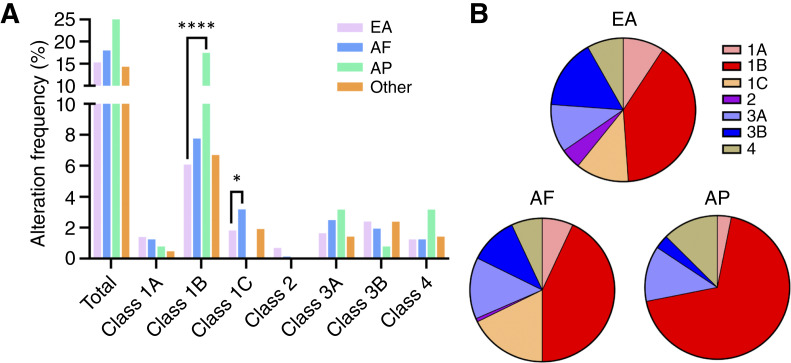
*FOXA1* alteration classes by race. **A, ***FOXA1* alteration classes are examined by race. **B,** The distribution of *FOXA1* alteration classes by race. *q value <0.05, **q value <0.01, ***q value < 0.001, ****q value < 0.0001.

We next examined associations with self-reported race, including European Americans (EA), African Americans (AF), and Asian/Pacific Islanders (AP; Fig. 6A). Strikingly, overall *FOXA1* alterations were present in 25.4% of AP patients with prostate cancer. Furthermore, compared with EA patients, AP and AF patients exhibited significant differences in the distribution of *FOXA1* alterations (Fig. 6B; Supplementary Table S11). For example, AP patients had significantly more class 1B (17.5% vs. 7.0%; *P* < 0.0001) with numerically increased class 3A (3.2% vs. 1.7%; *P* > 0.05.) alterations relative to EA patients. AF patients had significantly greater frequencies of class 1C alterations compared with EA patients (3.2% vs. 1.8%; *P* = 0.029).

## Discussion

Our study indicates that an aggregated classification of all pathogenic *FOXA1* mutations is not a suitable way to describe these tumors' molecular and clinical consequences. Importantly, the type of alteration (missense, in-frame, truncation, and amplification) and its structural consequences on the FKH domain can be used to define subsets of prostate tumors with distinct features. Among 5,014 prostate cancer cases, class 1B *FOXA1* alterations were the most prevalent, whereas the pathognomonic class 2 (R219) alterations were the rarest (but enriched in NEPC samples). Although these two subclasses both involve the FKH domain, our 3D structural analysis indicated that 1B and 2 residues occupy distinct protein surfaces. These were associated with distinct overall outcomes and sensitivity to first-generation ADT. Other than class 1B and 2 alterations, we also considered frameshift and truncating events concerning the FKH domain. This defined class 3A (in or before FKH) and 3B (after FKH) alterations, which were molecularly distinct and also had opposing clinical outcomes. As we found that *FOXA1* alterations were also modestly frequent in breast, bladder, and salivary cancers, subsets of which also express the AR (35–37), it is logical to test our classification scheme in nonprostatic cancers moving forward. Altogether, there are many indications in which the classification scheme presented here may warrant further investigation in prospective mechanistic and clinical studies.

In our analysis, AF and AP patients exhibited notably higher frequencies of specific classes of *FOXA1* alterations relative to EA patients. Astonishingly, ∼80 of the *FOXA1* alterations found in AP patients fell in class 1B and class 3A residues. A previous report indicated that 41% of prostate tumors from a Chinese cohort harbored *FOXA1* alterations (38). Although it is tempting to cross-compare our studies and the conclusions, the Caris POA cohort unfortunately does not include fine annotations of genetic ancestry; therefore, we are unable to specifically determine whether such patients are of Chinese descent as compared with other AP heritages. Furthermore, the Caris POA dataset includes tumors from centers within the United States. For such reasons, the AP patients may be affected by the socioeconomic factors, diets, and standard practices within the United States—all independent factors that may affect prostate cancer outcomes (39–42). Regardless, our study suggests that finer elements of race should be considered when evaluating patients who harbor *FOXA1* alterations moving forward.

Previous research groups have focused on understanding the consequences of *FOXA1* alterations on tumoral properties (8, 9). Perhaps the most notable agreement in the field is the indication that FKH domain alterations (which we have termed classes 1A, 1B, 2, and 3A) have distinct somatic landscapes and signaling properties with respect to WT. Here, we classified missense/in-frame FKH alterations as class 1A (non-Wing2) and 1B (involving the Wing2 region). *FOXA1* FKH domain alterations are thought to enhance AR signaling (9), and we found enriched hallmark androgen response in tumors with class 1A and 1B events. Adams and colleagues (8) were the first to indicate that R219 mutations, which we designate as class 2, often promote the transition to NEPC. This was mostly concordant with our findings, as we found that class 2 tumors were enriched in an NEPC transcriptional signature. It was the only class with a reduced hallmark androgen response based on the GSEA analysis. Although it is tempting to assign all class 2–related poor outcomes toward the development of NEPC, we surprisingly found that two-thirds of these cases were histologically acinar adenocarcinomas. This indicates that the class 2 *FOXA1* variants may be “hidden” in adenocarcinoma cases and are generally the indicators of poor prognosis among patients with metastatic disease.

Based on molecular profiling and TME analysis via quanTIseq, our findings indicate that *FOXA1* alterations may regulate tumor cell–extrinsic properties. Although prostate cancer is generally considered an immunologically “cold” cancer (43, 44), class 1C and 3B alterations had substantially greater frequencies (three- to fivefold) of TMB-high and MSI-high status as compared with WT, potentially selecting for tumors responsive to immune checkpoint inhibitors. Alternatively, these class 1C and 3B alterations may simply be bystanders of the hypermutation process. On the contrary, multiple hallmark inflammatory pathways and our profiling of the TME indicated that class 1B prostate cancers were immunologically “cold” compared with WT. Class 1B tumors also had reduced *TMPRSS2*–*ERG* fusions and fewer *TP53* alterations that may contribute to this immune suppressive state. Altogether, *FOXA1* alterations affect tumoral properties, disease progression, and the TME, all elements that should be considered in the development of novel therapeutics targeting these patients.

Overall, we have used the structural and functional information of the FOXA1 protein coupled with new clinical insights from a large clinically annotated database to assign a revised classification schema to *FOXA1* alterations. This approach, although conducted in this study in the context of prostate cancer, may potentially be more broadly applicable to additional driver genes in other human cancers. For example, p53, the protein product of *TP53*, is the most highly altered protein in human cancers. *TP53* mutations are mainly loss-of-function mutations, whereas mutations in certain residues are gain-of-function mutations (45, 46). Although p53 primarily regulates DNA repair, gain-of-function p53 mutations are associated with increased tumorigenic activities that drive metastasis, modulate the TME, or induce PD-L1 expression (47, 48). By analogy, class 1B and 2 *FOXA1* alterations are also associated with differences in the TME and in NEPC signaling as well as portending opposing clinical outcomes. The structural knowledge of p53 and FOXA1 may partially explain why we observe recurrent hotspot mutations in these genes, which may facilitate tumor homeostasis by enhancing, stabilizing, or prohibiting the native protein function. Altogether, we argue that appropriate structure-function knowledge should be included in clinical-grade genomic reports to inform prognosis and possibly influence therapeutic considerations. This requires fine knowledge and testable experimental models, as well as prospective studies in which relevant clinical outcomes are assessable.

### Study limitations

Our study had several limitations with respect to measuring patient outcomes, treatment response, and pathologic annotations of adenocarcinoma versus NEPC. The biopsy location (prostate or metastasis) does not reflect the disease stage or tumor grade at diagnosis, and this patient population is heterogeneous. All annotations of death in survival analyses have been inferred from insurance claim data. Therefore, OS analysis should be considered exploratory and requires further validation in future studies. Tumor biopsies were performed at various time points in relation to the treatments, and we are unable to access information regarding the line of systemic therapy, time of metastasis, or indicators that the prostate cancer is locally advanced versus metastatic at initial diagnosis or at the initiation of first systemic therapy. We are unable to perform multivariate analyses to determine the independent prognostic impact of *FOXA1* alteration classes. As such, only randomized trials with the interrogation of *FOXA1* status would be able to determine whether this could be used as a predictive (treatment selection) biomarker. Therefore, the responses to specific therapies reported here should be considered only prognostic rather than predictive. With respect to histologic annotation, although pathologists at Caris Life Sciences have determined that specific cases of adenocarcinomas harboring R219 mutations did not exhibit morphologic evidence of neuroendocrine differentiation upon second histopathologic review, there is a possibility that undetected NEPC cells were not properly identified because of an inability to assess neuroendocrine IHC markers, as well as issues related to evaluating depleted samples obtained from larger tumors. Finally, the immune deconvolution of these tumor samples was inferred from bulk RNA sequencing rather than sequencing of single immune cells. Additionally, we organized the data based on self-reported race, whereby certain patients were uncharacterized and excluded from analysis.

### Conclusions

In summary, we show that *FOXA1* alterations have different molecular and clinical implications when considered by class, suggesting the utility of reporting them separately in clinical-grade genomic tests. We have proposed a revised clinical-molecular classification scheme, showing that the different classes of *FOXA1* alterations have distinct clinical outcomes, different immune milieus, and additional potential precision therapy targets. However, this study falls short of a detailed clinical characterization of the prognostic impact of *FOXA1* alterations in prostate cancer because of significant limitations in the clinical data captured and the inability to adjust survival outcomes by metastatic or nonmetastatic status as well as lines of systemic therapy. To confirm the clinical utility of our classification scheme, future studies should carefully examine how these *FOXA1* alteration classes govern outcomes in clinically relevant settings, including in primary prostate cancer, metastatic hormone-sensitive disease, and metastatic castration-resistant disease.

## Supplementary Material

Supplementary Figure 1-10 Supplementary Figures 1-10

Supplementary Table 1-11 Supplementary Tables 1-11

## References

[1] Sung H , Ferlay J, Siegel RL, Laversanne M, Soerjomataram I, Jemal A, . Global cancer statistics 2020: GLOBOCAN estimates of incidence and mortality worldwide for 36 cancers in 185 countries. CA Cancer J Clin 2021;71:209–49. 33538338 10.3322/caac.21660

[2] Dai C , Heemers H, Sharifi N. Androgen signaling in prostate cancer. Cold Spring Harb Perspect Med 2017;7:a030452. 28389515 10.1101/cshperspect.a030452 PMC5580512

[3] Pomerantz MM , Li F, Takeda DY, Lenci R, Chonkar A, Chabot M, . The androgen receptor cistrome is extensively reprogrammed in human prostate tumorigenesis. Nat Genet 2015;47:1346–51. 26457646 10.1038/ng.3419 PMC4707683

[4] Jozwik KM , Carroll JS. Pioneer factors in hormone-dependent cancers. Nat Rev Cancer 2012;12:381–5. 22555282 10.1038/nrc3263

[5] Teng M , Zhou S, Cai C, Lupien M, He HH. Pioneer of prostate cancer: past, present and the future of FOXA1. Protein Cell 2021;12:29–38. 32946061 10.1007/s13238-020-00786-8 PMC7815845

[6] Baca SC , Takeda DY, Seo J-H, Hwang J, Ku SY, Arafeh R, . Reprogramming of the FOXA1 cistrome in treatment-emergent neuroendocrine prostate cancer. Nat Commun 2021;12:1979. 33785741 10.1038/s41467-021-22139-7 PMC8010057

[7] Pomerantz MM , Qiu X, Zhu Y, Takeda DY, Pan W, Baca SC, . Prostate cancer reactivates developmental epigenomic programs during metastatic progression. Nat Genet 2020;52:790–9. 32690948 10.1038/s41588-020-0664-8 PMC10007911

[8] Adams EJ , Karthaus WR, Hoover E, Liu D, Gruet A, Zhang Z, . FOXA1 mutations alter pioneering activity, differentiation and prostate cancer phenotypes. Nature 2019;571:408–12. 31243370 10.1038/s41586-019-1318-9 PMC6661172

[9] Parolia A , Cieslik M, Chu S-C, Xiao L, Ouchi T, Zhang Y, . Distinct structural classes of activating FOXA1 alterations in advanced prostate cancer. Nature 2019;571:413–18. 31243372 10.1038/s41586-019-1347-4 PMC6661908

[10] Imamura Y , Sakamoto S, Endo T, Utsumi T, Fuse M, Suyama T, . FOXA1 promotes tumor progression in prostate cancer via the insulin-like growth factor binding protein 3 pathway. PLoS One 2012;7:e42456. 22879989 10.1371/journal.pone.0042456 PMC3411739

[11] Sahu B , Laakso M, Ovaska K, Mirtti T, Lundin J, Rannikko A, . Dual role of FoxA1 in androgen receptor binding to chromatin, androgen signalling and prostate cancer. EMBO J 2011;30:3962–76. 21915096 10.1038/emboj.2011.328 PMC3209787

[12] Abida W , Cyrta J, Heller G, Prandi D, Armenia J, Coleman I, . Genomic correlates of clinical outcome in advanced prostate cancer. Proc Natl Acad Sci U S A 2019;116:11428–36. 31061129 10.1073/pnas.1902651116 PMC6561293

[13] Robinson D , Van Allen EM, Wu Y-M, Schultz N, Lonigro RJ, Mosquera J-M, . Integrative clinical genomics of advanced prostate cancer. Cell 2015;162:454. 28843286 10.1016/j.cell.2015.06.053

[14] Barbieri CE , Baca SC, Lawrence MS, Demichelis F, Blattner M, Theurillat J-P, . Exome sequencing identifies recurrent SPOP, FOXA1 and MED12 mutations in prostate cancer. Nat Genet 2012;44:685–9. 22610119 10.1038/ng.2279 PMC3673022

[15] Cirillo LA , Zaret KS. Specific interactions of the wing domains of FOXA1 transcription factor with DNA. J Mol Biol 2007;366:720–4. 17189638 10.1016/j.jmb.2006.11.087 PMC1793999

[16] Dai S , Qu L, Li J, Chen Y. Toward a mechanistic understanding of DNA binding by forkhead transcription factors and its perturbation by pathogenic mutations. Nucleic Acids Res 2021;49:10235–49. 34551426 10.1093/nar/gkab807 PMC8501956

[17] Avila M , Meric-Bernstam F. Next-generation sequencing for the general cancer patient. Clin Adv Hematol Oncol 2019;17:447–54. 31449513 PMC6739831

[18] Abraham JP , Magee D, Cremolini C, Antoniotti C, Halbert DD, Xiu J, . Clinical validation of a machine-learning-derived signature predictive of outcomes from first-line oxaliplatin-based chemotherapy in advanced colorectal cancer. Clin Cancer Res 2021;27:1174–83. 33293373 10.1158/1078-0432.CCR-20-3286

[19] Hwang J , Shi X, Elliott A, Arnoff TE, McGrath J, Xiu J, . Metastatic prostate cancers with BRCA2 versus ATM mutations exhibit divergent molecular features and clinical outcomes. Clin Cancer Res 2023;29:2702–13. 37126020 10.1158/1078-0432.CCR-22-3394 PMC12211896

[20] Epstein JI , Amin MB, Beltran H, Lotan TL, Mosquera J-M, Reuter VE, . Proposed morphologic classification of prostate cancer with neuroendocrine differentiation. Am J Surg Pathol 2014;38:756–67. 24705311 10.1097/PAS.0000000000000208 PMC4112087

[21] Silk M , Petrovski S, Ascher DB. MTR-Viewer: identifying regions within genes under purifying selection. Nucleic Acids Res 2019;47:W121–6. 31170280 10.1093/nar/gkz457 PMC6602522

[22] Benjamini Y , Drai D, Elmer G, Kafkafi N, Golani I. Controlling the false discovery rate in behavior genetics research. Behav Brain Res 2001;125:279–84. 11682119 10.1016/s0166-4328(01)00297-2

[23] Choi Y , Luo Y, Lee S, Jin H, Yoon H-J, Hahn Y, . FOXL2 and FOXA1 cooperatively assemble on the TP53 promoter in alternative dimer configurations. Nucleic Acids Res 2022;50:8929–46. 35920317 10.1093/nar/gkac673 PMC9410875

[24] Newman JA , Aitkenhead H, Gavard AE, Rota IA, Handel AE, Hollander GA, . The crystal structure of human forkhead box N1 in complex with DNA reveals the structural basis for forkhead box family specificity. J Biol Chem 2020;295:2948–58. 31914405 10.1074/jbc.RA119.010365 PMC7062188

[25] Schymkowitz J , Borg J, Stricher F, Nys R, Rousseau F, Serrano L. The FoldX web server: an online force field. Nucleic Acids Res 2005;33:W382–388. 15980494 10.1093/nar/gki387 PMC1160148

[26] UniProt Consortium . UniProt: the universal protein knowledgebase in 2023. Nucleic Acids Res 2023;51:D523–31. 36408920 10.1093/nar/gkac1052 PMC9825514

[27] Jumper J , Evans R, Pritzel A, Green T, Figurnov M, Ronneberger O, . Highly accurate protein structure prediction with AlphaFold. Nature 2021;596:583–9. 34265844 10.1038/s41586-021-03819-2 PMC8371605

[28] Ayers M , Lunceford J, Nebozhyn M, Murphy E, Loboda A, Kaufman DR, . IFN-gamma-related mRNA profile predicts clinical response to PD-1 blockade. J Clin Invest 2017;127:2930–40. 28650338 10.1172/JCI91190 PMC5531419

[29] Bao R , Stapor D, Luke JJ. Molecular correlates and therapeutic targets in T cell-inflamed versus non-T cell-inflamed tumors across cancer types. Genome Med 2020;12:90. 33106165 10.1186/s13073-020-00787-6 PMC7590690

[30] Beltran H , Prandi D, Mosquera JM, Benelli M, Puca L, Cyrta J, . Divergent clonal evolution of castration-resistant neuroendocrine prostate cancer. Nat Med 2016;22:298–305. 26855148 10.1038/nm.4045 PMC4777652

[31] Subramanian A , Tamayo P, Mootha VK, Mukherjee S, Ebert BL, Gillette MA, . Gene set enrichment analysis: a knowledge-based approach for interpreting genome-wide expression profiles. Proc Natl Acad Sci U S A 2005;102:15545–50. 16199517 10.1073/pnas.0506580102 PMC1239896

[32] Finotello F , Mayer C, Plattner C, Laschober G, Rieder D, Hackl H, . Molecular and pharmacological modulators of the tumor immune contexture revealed by deconvolution of RNA-seq data. Genome Med 2019;11:34. 31126321 10.1186/s13073-019-0638-6 PMC6534875

[33] Abida W , Armenia J, Gopalan A, Brennan R, Walsh M, Barron D, . Prospective genomic profiling of prostate cancer across disease states reveals germline and somatic alterations that may affect clinical decision making. JCO Precis Oncol 2017;2017:PO.17.00029. 28825054 10.1200/PO.17.00029 PMC5558263

[34] Antonarakis ES , Shaukat F, Isaacsson Velho P, Kaur H, Shenderov E, Pardoll DM, . Clinical features and therapeutic outcomes in men with advanced prostate cancer and DNA mismatch repair gene mutations. Eur Urol 2019;75:378–82. 30337059 10.1016/j.eururo.2018.10.009 PMC6377812

[35] Anestis A , Zoi I, Papavassiliou AG, Karamouzis MV. Androgen receptor in breast cancer-clinical and preclinical research insights. Molecules 2020;25:358. 31952272 10.3390/molecules25020358 PMC7024330

[36] Li P , Chen J, Miyamoto H. Androgen receptor signaling in bladder cancer. Cancers (Basel) 2017;9:20. 28241422 10.3390/cancers9020020 PMC5332943

[37] Xu B , Dogan S, Haroon Al Rasheed MR, Ghossein R, Katabi N. Androgen receptor immunohistochemistry in salivary duct carcinoma: a retrospective study of 188 cases focusing on tumoral heterogeneity and temporal concordance. Hum Pathol 2019;93:30–6. 31430492 10.1016/j.humpath.2019.08.007 PMC6937722

[38] Li J , Xu C, Lee HJ, Ren S, Zi X, Zhang Z, . A genomic and epigenomic atlas of prostate cancer in Asian populations. Nature 2020;580:93–9. 32238934 10.1038/s41586-020-2135-x

[39] Coughlin SS . A review of social determinants of prostate cancer risk, stage, and survival. Prostate Int 2020;8:49–54. 32647640 10.1016/j.prnil.2019.08.001 PMC7335972

[40] Kelly BD , Perera M, Bolton DM, Papa N. Social determinants of health: does socioeconomic status affect access to staging imaging for men with prostate cancer. Prostate Cancer Prostatic Dis 2023;26:429–31. 35169274 10.1038/s41391-022-00508-7 PMC9376196

[41] Key TJ , Silcocks PB, Davey GK, Appleby PN, Bishop DT. A case-control study of diet and prostate cancer. Br J Cancer 1997;76:678–87. 9303371 10.1038/bjc.1997.445 PMC2228001

[42] Matsushita M , Fujita K, Nonomura N. Influence of diet and nutrition on prostate cancer. Int J Mol Sci 2020;21:1447. 32093338 10.3390/ijms21041447 PMC7073095

[43] Antonarakis ES , Park SH, Goh JC, Shin SJ, Lee JL, Mehra N, . Pembrolizumab plus olaparib for patients with previously treated and biomarker-unselected metastatic castration-resistant prostate cancer: the randomized, open-label, phase III KEYLYNK-010 trial. J Clin Oncol 2023;41:3839–50. 37290035 10.1200/JCO.23.00233 PMC10419579

[44] Antonarakis ES , Piulats JM, Gross-Goupil M, Goh J, Ojamaa K, Hoimes CJ, . Pembrolizumab for treatment-refractory metastatic castration-resistant prostate cancer: multicohort, open-label phase II KEYNOTE-199 study. J Clin Oncol 2020;38:395–405. 31774688 10.1200/JCO.19.01638 PMC7186583

[45] Baugh EH , Ke H, Levine AJ, Bonneau RA, Chan CS. Why are there hotspot mutations in the TP53 gene in human cancers? Cell Death Differ 2018;25:154–60. 29099487 10.1038/cdd.2017.180 PMC5729536

[46] Olivier M , Hollstein M, Hainaut P. TP53 mutations in human cancers: origins, consequences, and clinical use. Cold Spring Harb Perspect Biol 2010;2:a001008. 20182602 10.1101/cshperspect.a001008 PMC2827900

[47] Alvarado-Ortiz E , de la Cruz-López KG, Becerril-Rico J, Sarabia-Sánchez MA, Ortiz-Sánchez E, García-Carrancá A. Mutant p53 gain-of-function: role in cancer development, progression, and therapeutic approaches. Front Cell Dev Biol 2020;8:607670. 33644030 10.3389/fcell.2020.607670 PMC7905058

[48] Kennedy MC , Lowe SW. Mutant p53: it’s not all one and the same. Cell Death Differ 2022;29:983–7. 35361963 10.1038/s41418-022-00989-y PMC9090915

