# Supplementary material for: Structurally Oriented Classification of FOXA1 Alterations Identifies Prostate Cancers with Opposing Clinical Outcomes and Distinct Molecular and Immunologic Subtypes
Source: Clin Cancer Res. Author manuscript; Available in PMC 2025 Mar 4. (PMC11873805; doi:10.1158/1078-0432.CCR-24-3471)
Supplement: Supplementary Figures [file NIHMS2056757-supplement-Supplementary_Figures.pdf]

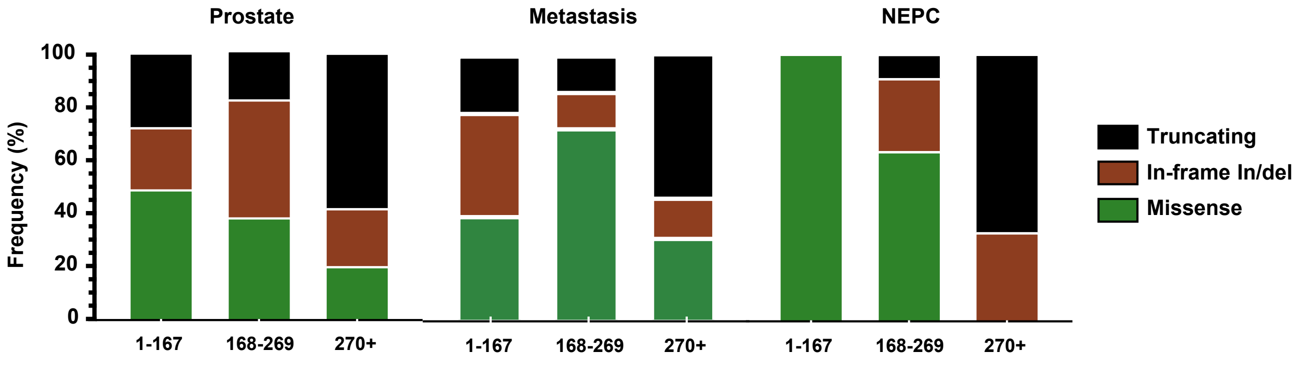

Supplementary Figure 1. *FOXA1* alterations by amino acid sequence and Forkhead (FKH) domain. Stacked bar graphs showing distribution of the type of *FOXA1* alterations, missense (green), in-frame indels (brown), and predicted truncations (black) among three distinct regions of the gene (before FKH domain, in FKH domain, and after FKH domain).

A

Primary Tumors

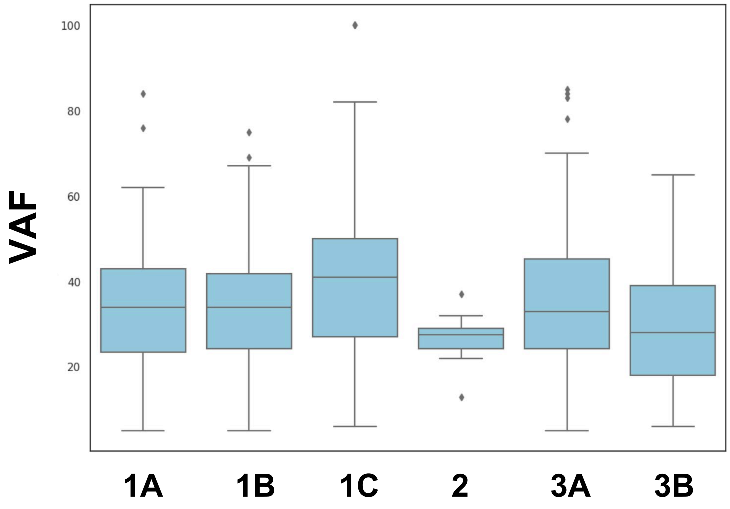

Class	Median VAF
1A	34.0
1B	34.0
1C	41.0
2	27.5
3A	33.0
3B	28.0

B

Metastatic Tumors

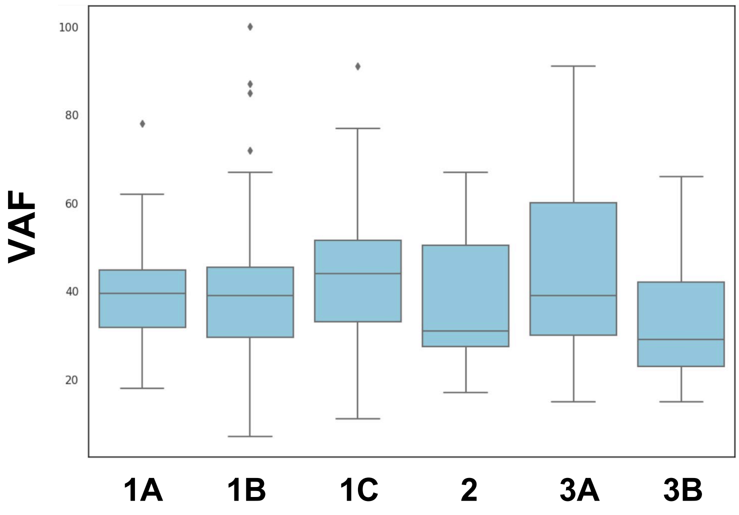

Class	Median VAF
1A	37.0
1B	39.0
1C	44.0
2	31.0
3A	39.0
3B	30.0

Supplementary Figure 2. Variant allele frequency (VAF) of each *FOXA1* alteration class. Boxplots showing VAF of each *FOXA1* subclass in prostate **(A)** and metastatic cohorts **(B)**

A

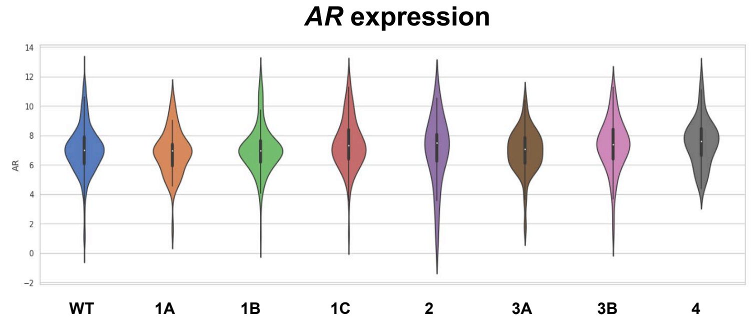

	Median	q-value
WT	7.00	
1A	6.97	0.73
1B	6.95	0.74
1C	7.31	0.11
2	7.49	0.74
3A	7.05	0.88
3B	7.38	0.07
4	7.58	0.07

B

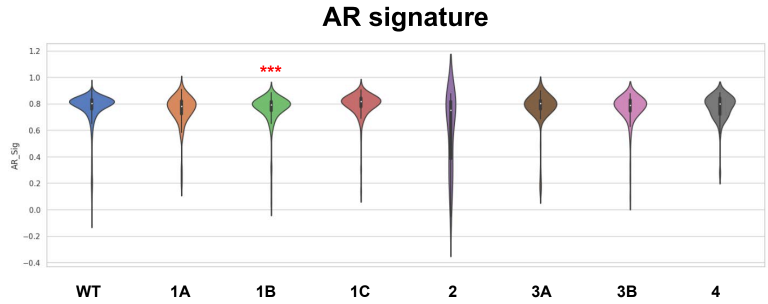

	Median	q-value
WT	0.80	
1A	0.78	0.14
1B	0.79	0.0004
1C	0.82	0.09
2	0.75	0.09
3A	0.80	0.78
3B	0.79	0.23
4	0.80	0.85

C

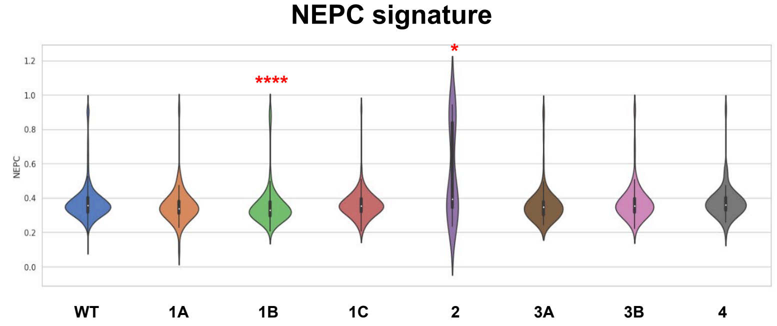

	Median	q-value
WT	0.36	
1A	0.34	0.12
1B	0.33	1.9E-10
1C	0.35	0.60
2	0.39	0.02
3A	0.35	0.06
3B	0.35	0.93
4	0.36	0.60

Supplemental Figure 3. Transcriptional associations by *FOXA1* alteration class. Violin plots showing **(A)** AR mRNA expression, **(B)** AR signature score, and **(C)** NEPC signature score based on alteration class. The median is reflected by horizontal white lines. q-values are established based on comparisons to wild-type (WT). * q-value <0.05, ** q-value <0.01, *** q-value < 0.001, **** q-value < 0.0001

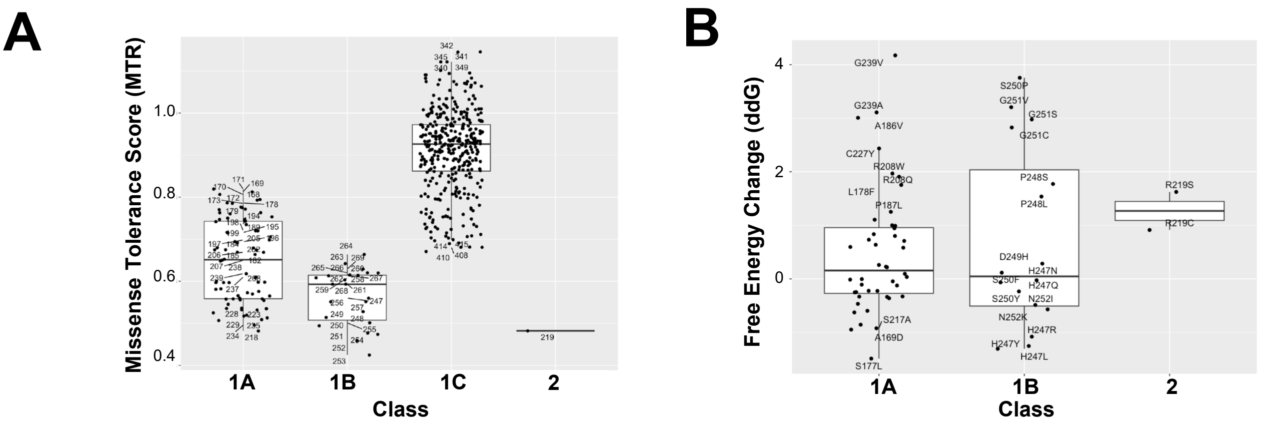

Supplementary Figure 4. *FOXA1* alterations and impact on protein structure. (A) Boxplot of each MTR score based on *FOXA1* alteration class. **(B)** *In silico* mutagenic analysis of FOXA1 stability, represented by the change in Gibbs free energy (ddG) for variants identified in clinical samples.

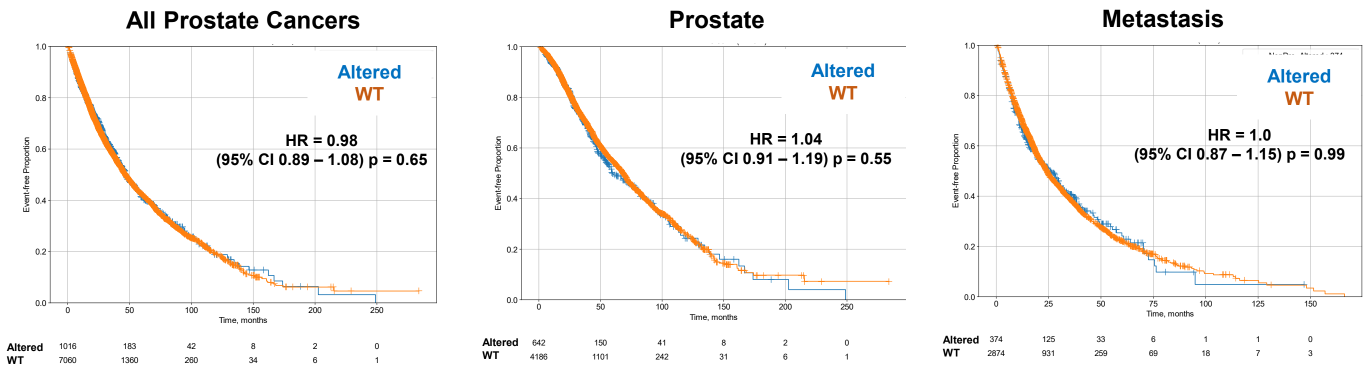

Supplementary Figure 5. Overall survival (OS) or *FOXA1* alterations in aggregate. OS analysis is depicted through Kaplan-Meier curves that compare *FOXA1* altered tumors to wild-type (WT). The analysis is done based on all prostate tumor samples, or if the tissue was obtained from the prostate or metastatic sites.

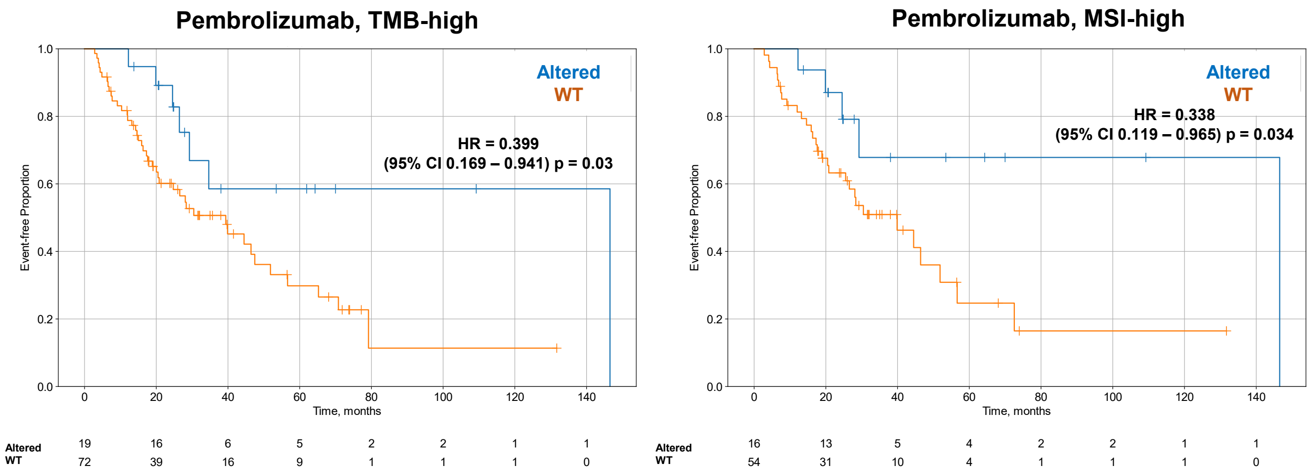

Supplementary Figure 6. Outcomes in pembrolizumab based on *FOXA1* alterations in aggregate. Kaplan Meier analysis was performed to evaluate differences in outcomes in TMB-high and MSI-high patients, respectively, according to *FOXA1* co-alteration status.

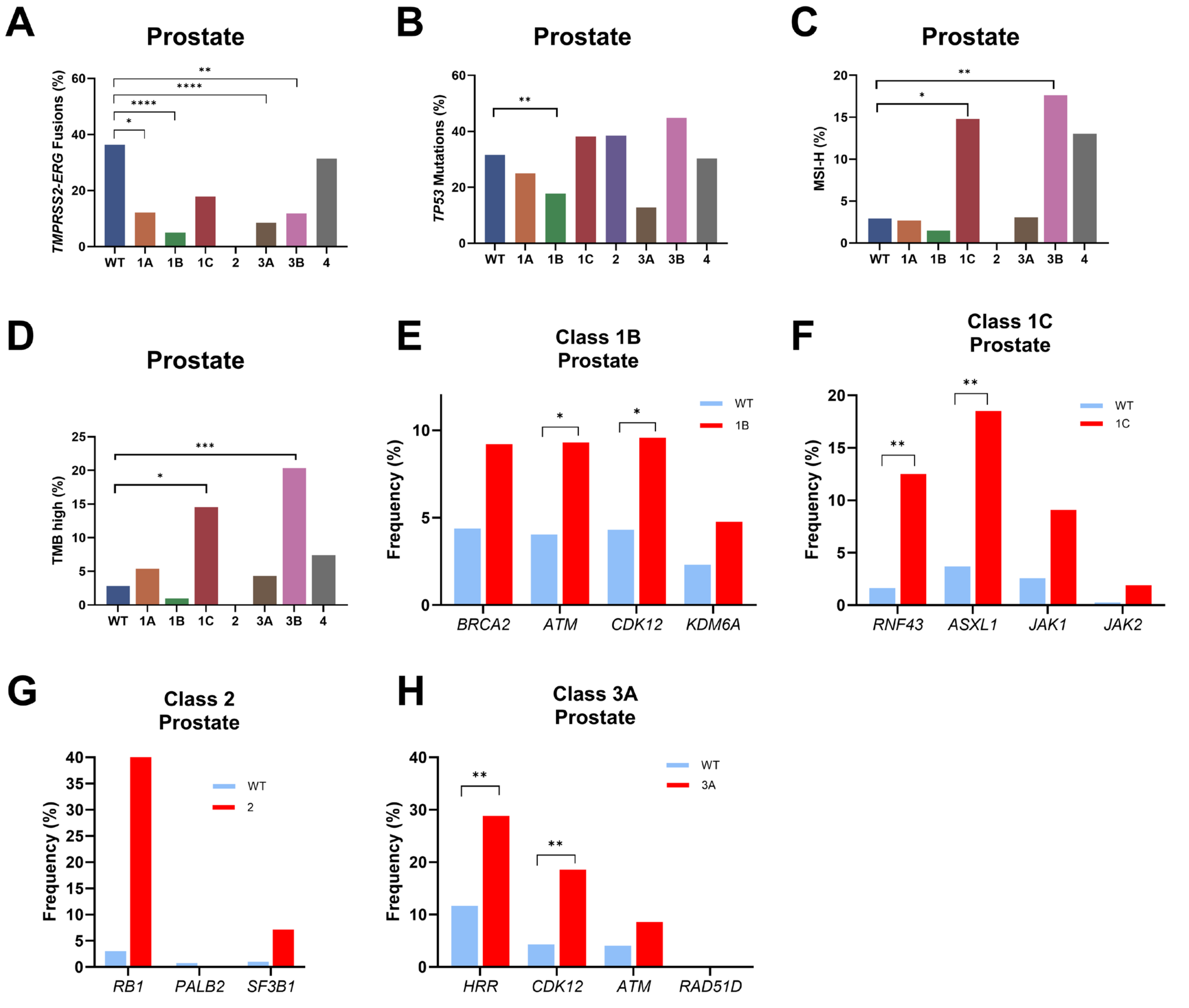

Supplementary Figure 7. Molecular associations by *FOXA1* alteration class in primary tumor biopsies. For all samples from prostate biopsies, we depict the percentages of **(A)** *TMPRSS2-ERG* fusions, **(B)** *TP53* mutations, **(C)** MSI-high status, and **(D)** TMB-high status based on *FOXA1* alteration classes. Percentages of select genetic alterations comparing wild-type and **(E)** class 1B, **(F)** class 1C, **(G)** class 2, **(H)** and class 3A. * q-value <0.05, ** q-value <0.01, *** q-value < 0.001, **** q-value < 0.0001.

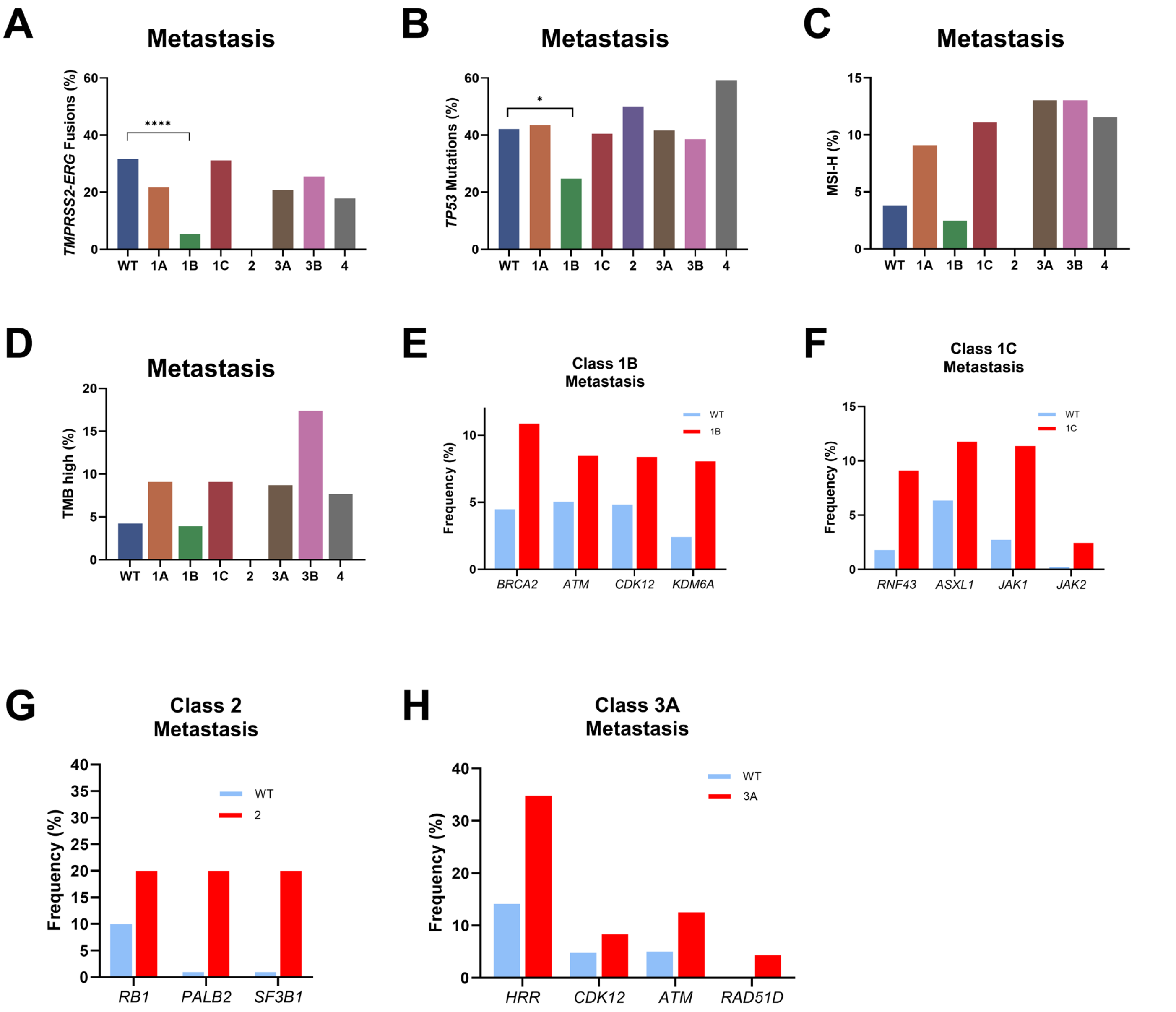

Supplementary Figure 8. Molecular associations by *FOXA1* alteration class in metastatic tumor biopsies. For all biopsies from metastatic sites, we depict the percentages of **(A)** *TMPRSS2-ERG* fusions, **(B)** *TP53* mutations, **(C)** MSI-high status, and **(D)** TMB-high status based on *FOXA1* alteration classes. Percentages of select genetic alterations comparing wild-type and **(E)** class 1B, **(F)** class 1C, **(G)** class 2, **(H)** and class 3A. * q-value <0.05, ** q-value <0.01, *** q-value < 0.001, **** q-value < 0.0001.

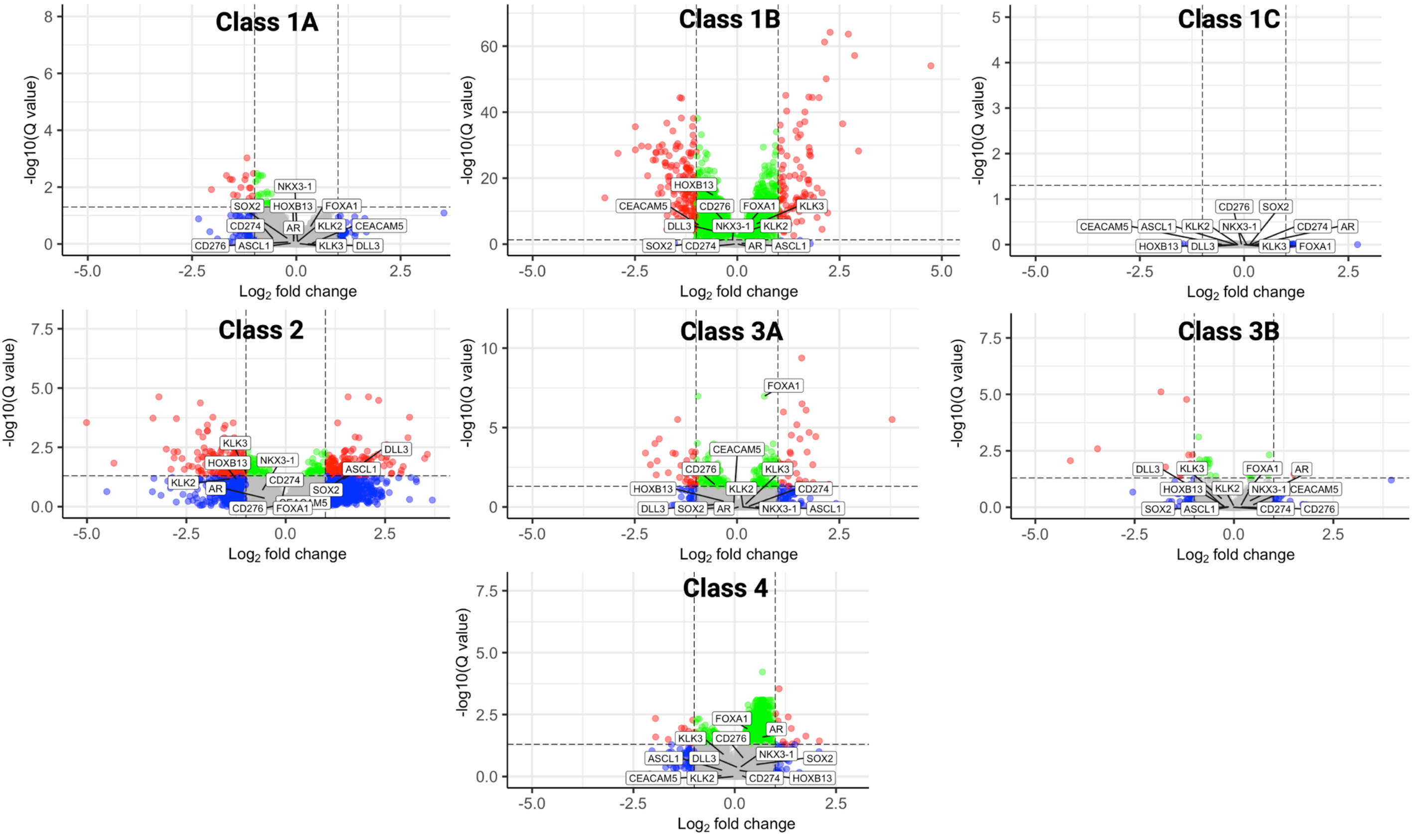

Supplementary Figure 9. Depiction of differentially expressed transcripts by *FOXA1* alterations. Whole transcriptomes were analyzed in which the differences between each class of *FOXA1* alteration is shown as compared to wild type (WT) samples. Key genes are shown based on their known association with AR signaling (*AR*, *KLK3/PSA*, *KLK3*, *HOXB13*, *FOXA1*, *NKX3-1*), NEPC phenotype (*ASCL1*, *SOX2*), and specific cell surface targets (*CD274/PD-L1*, *CD276/B7-H3*, *CEACAM5*, *DLL3*). Genes exhibiting significant (but less than 2-fold) changes are shown in green. Significant genes that are differentially expressed by more than 2-fold are shown in red. Genes that are not significant but exhibited more than 2-fold changes are shown in blue.

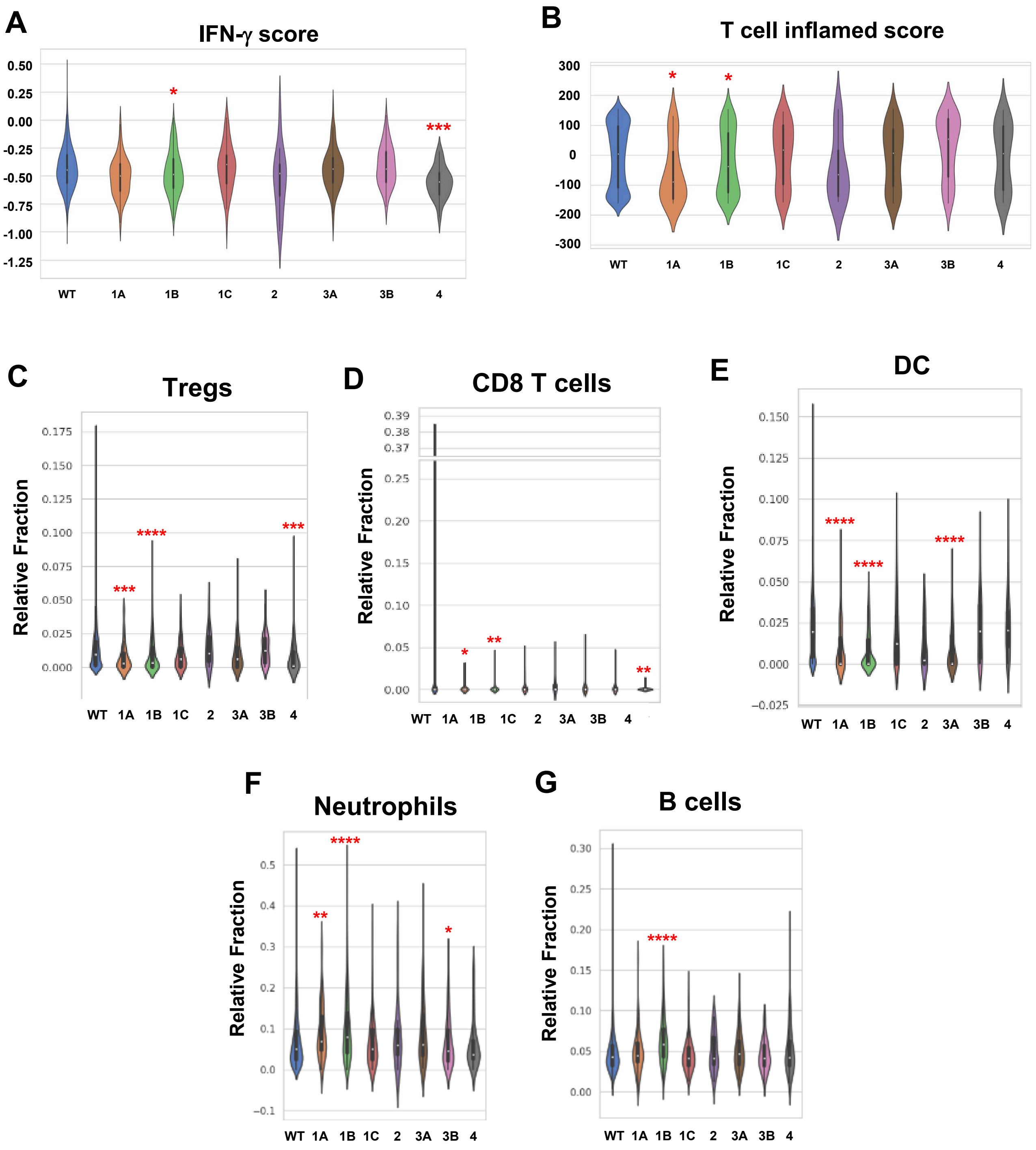

Supplementary Figure 10. Alterations in the tumor microenvironment by *FOXA1* alteration class. Violin plots showing **(A)** IFN-γ score and **(B)** T-cell inflamed score in prostate tumor samples. Based on quanTIseq, immune cell fractions are shown by class for **(C)** T regs, **(D)** CD8 T-cells, **(E)** Dendritic cell, **(F)** Neutrophils, and **(G)** B-cells.
